# Nanozymes: bridging nanoscale characteristics and catalytic mechanisms for advanced biomedical applications

**DOI:** 10.1080/14756366.2026.2690829

**Published:** 2026-06-22

**Authors:** Yunqin Sun, Yunchang Yang, Xinyao Kang, Yaofeng Wang

**Affiliations:** ^a^ North Henan Medical University, Xinxiang, China; ^b^ Henan university, Kaifeng, China

**Keywords:** Nanozymes, enzyme mimic, disease diagnosis, biosensor, clinical translation

## Abstract

Nanozymes—nanomaterials with intrinsic enzyme-like activity—have emerged as a transformative paradigm in biomedicine. Their catalytic proficiency is fundamentally dictated by unique nanoscale characteristics, particularly tuneable surface defects and geometric configurations. This review critically re-evaluates nanozyme catalytic mechanisms, precisely categorising them into reactive oxygen species (ROS)-generating pathways and electron-transfer processes. We highlight recent structural breakthroughs, emphasising the evolution from conventional nanoparticles to single-atom and dual-atom nanozymes (SANs/DANs) that exhibit unprecedented reaction kinetics. Furthermore, we synthesise their advanced applications across in vitro diagnostics, synergistic tumour therapy, antibacterial interventions, and regenerative medicine. Finally, we address current clinical translational bottlenecks and envision how artificial intelligence (AI)-guided rational design will shape the field’s future. By bridging fundamental physical chemistry with clinical utility, this review provides a definitive mechanistic roadmap for deploying nanozymes in precision nanomedicine.

## Graphical Abstract

**Figure:**
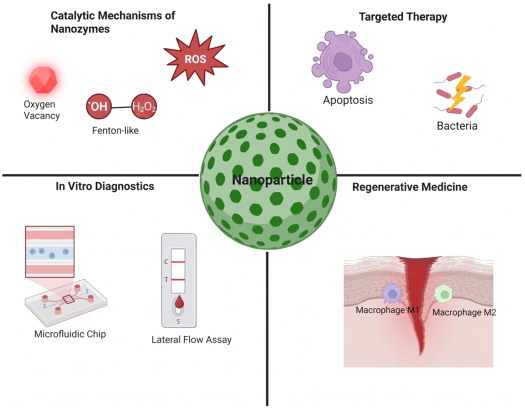

Schematic illustration of nanozymes bridging fundamental catalytic mechanisms and advanced biomedical applications. The central nanoparticle emphasises the critical role of nanoscale characteristics and surface engineering in dictating intrinsic enzyme-like activity. The surrounding panels comprehensively outline their rationally designed pathways and versatile clinical potential, encompassing highly sensitive in vitro diagnostics, targeted therapeutics (tumour apoptosis and antibacterial strategies), and emerging roles in regenerative medicine (e.g., promoting tissue repair via M1-to-M2 macrophage repolarization).

## Introduction

Nanozymes refer to nanomaterials with intrinsic enzyme-like catalytic activities. Since the concept was first introduced in 2007, the field has experienced explosive growth^1^. Early studies fundamentally defined nanozymes as nanomaterials possessing intrinsic enzyme-like catalytic activity. However, driven by rapid advancements in materials science, this definition has significantly expanded. Currently, nanozymes are recognised as sophisticated nanostructures capable of rationally mimicking the geometric and electronic coordination environments of natural enzymes’ active sites^2^. Unlike natural enzymes, which are frequently constrained by poor environmental stability, high production costs, and complex purification processes, nanozymes offer exceptional thermodynamic robustness, highly tuneable catalytic kinetics, and versatile surface functionalization capabilities.

Despite the rapid advancements, a comprehensive understanding of how nanoscale characteristics govern their catalytic behaviour remains fragmented. Therefore, this review aims to systematically summarise the fundamental theory of nanozymes from a nanoscale perspective. We critically evaluate their catalytic mechanisms, highlight recent structural breakthroughs such as atomic-level nanozymes, and provide an up-to-date synthesis of their applications in disease diagnosis and therapy. Finally, we discuss current translational challenges and envision the integration of artificial intelligence in shaping the future of precision nanomedicine.

## Fundamental theory and structural evolution of nanozymes

A fundamental understanding of nanozymes requires integrating their compositional classification with their structural evolution towards atomically precise catalytic systems. In this section, we first define nanozymes from a nanoscale perspective and then systematically categorise them based on composition, followed by a discussion of their evolution towards advanced atomic-level architectures.

### The fundamental theory and evolutionary landscape of nanozymes

This section establishes the theoretical foundation of nanozyme catalysis from first principles. We begin by elucidating the nanoscale physicochemical origins that give rise to enzyme-like activity, then introduce a unified two-dimensional classification paradigm, and finally trace the structural evolution of nanozymes from conventional nanoparticles to atomically precise single-atom and dual-atom architectures.

#### Nanoscale origin of catalytic activity: a physicochemical perspective

The enzyme-like behaviour of nanozymes is not an incidental property but is fundamentally dictated by two interconnected nanoscale phenomena: surface electronic effects and finite size effects. Unlike bulk catalysts, nanomaterials possess an exceptionally high proportion of under-coordinated surface atoms, generating a dense population of metastable active sites. This endows nanozymes with an unusually high surface energy that facilitates the chemisorption and subsequent activation of reactant molecules. Simultaneously, quantum confinement alters the density of electronic states near the Fermi level, directly tuning the redox potential and charge transfer kinetics of the nanomaterial. Consequently, the catalytic properties of a nanozyme are not solely determined by its elemental composition; they arise from a complex interplay of surface crystallography (exposed facets, corners, and edges), defect chemistry (oxygen/sulphur vacancies), and the resulting electronic band structure alignment with the frontier molecular orbitals of substrates. Recognising these nanoscale origins provides the theoretical basis for the rational engineering strategies—from heteroatom doping to single-atom anchoring—discussed throughout this review^3^^,^^4^.

#### A two-dimensional classification paradigm

To establish a consistent and navigable framework, nanozymes are classified along two orthogonal dimensions: material chemistry and enzymological function. The material chemistry dimension traces the structural evolution from bulk compositions to atomic precision, encompassing metal/metal oxide-based, carbon-based, metal–organic framework (MOF)-based, and the emerging atomically precise architectures including single-atom nanozymes (SANs) and dual-atom nanozymes (DANs). The enzymological function dimension is defined by the specific type of catalytic reaction, predominantly oxidoreductase, hydrolase, and lyase activities. Crucially, these two dimensions are not mutually exclusive but are fully orthogonal; for instance, a Fe–N–C SAN (material chemistry axis) is simultaneously a potent peroxidase mimic (enzymological function axis). This dual-axis lens resolves the apparent discrepancy in category counts between figures and tables in the subsequent sections, and will be consistently employed to integrate diverse nanozyme systems into a coherent narrative.

#### Material chemistry dimension: from bulk to atomic precision

The definition of nanozymes has fundamentally evolved to emphasise their unique nanoscale characteristics—such as quantum confinement, ultra-high surface-to-volume ratios, and highly tuneable surface energies^2^. Unlike bulk catalysts, these finite size effects dictate their unique geometric and electronic structures, enabling them to rationally mimic the active sites of natural enzymes^1^. Along the material chemistry axis, nanozymes are primarily categorised into metal-based, carbon-based, and metal-organic framework (MOF)-based nanomaterials. Metal-based nanozymes, including transition metal oxides like Fe_3_O_4_ and CeO_2_, historically dominate the field due to their excellent stability and inherent redox cycling capabilities (e.g., Fe^2+^/Fe³^+^)^5^. Carbon-based nanozymes, such as graphene quantum dots and carbon nanotubes, derive their catalytic activity predominantly from surface defects and precise heteroatom doping^6^. MOF-based nanozymes achieve enzyme-like catalysis through highly designable synergistic interactions between defined metal nodes and organic ligands^7^. Additionally, transition metal sulphide-based nanozymes (e.g. CuCo_2_S_4_ and FeS_2_) have emerged as potent candidates, showing significant potential in biosensing and photothermal tumour therapy^8^. While the above categories encompass the majority of early nanozyme research, nanozyme development has undergone a clear structural evolution towards increasing precision and efficiency. Early nanozymes were primarily based on bulk nanomaterials and nanoparticles, where catalytic activity was governed by surface defects and exposed crystal facets. Although these systems demonstrated enzyme-like behaviour, their active sites were often heterogeneous and poorly defined.

To overcome these limitations, recent structural engineering has shifted towards optimising atomic utilisation and precision, marking the evolution from conventional nanoparticles to single-atom nanozymes (SANs) and dual-atom nanozymes (DANs)^2^^,^^9^. SANs feature isolated metal atoms (e.g., Fe, Cu, Pd) uniformly anchored on supportive substrates, typically nitrogen-doped carbon networks (M–N–C). This advanced architecture maximises atom utilisation to nearly 100% and closely replicates the geometric and electronic coordination environments of natural metalloenzymes (e.g., the haem group in porphyrin rings). For example, structurally defined Fe–N–C SANs have demonstrated unprecedented peroxidase-mimicking activity that significantly surpasses that of natural horseradish peroxidase (HRP)^10^.

**Figure 1.**
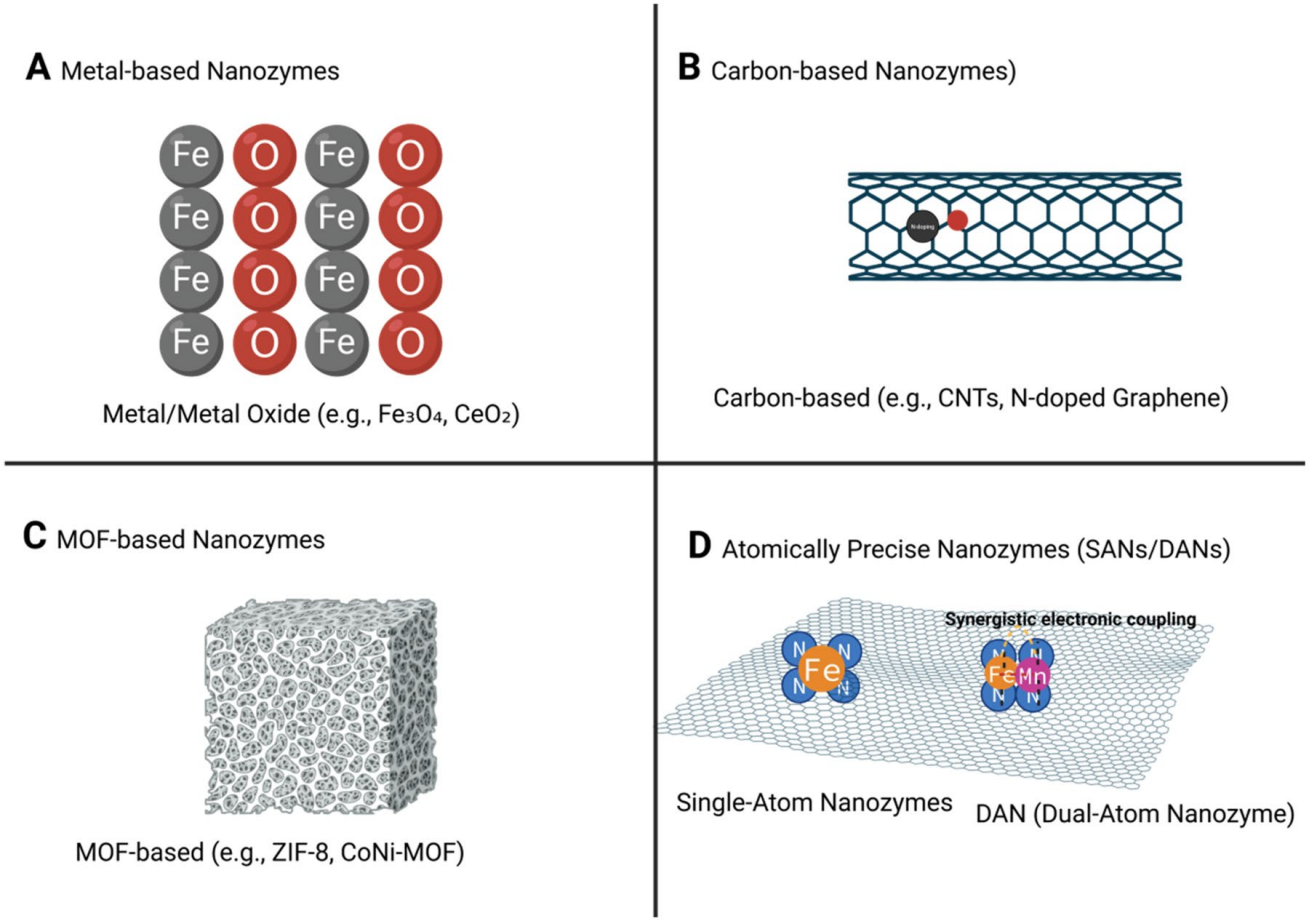
Representative structural configurations and the evolutionary trajectory of primary nanozyme categories. (A) Metal and metal oxide-based nanozymes (e.g., Fe_3_O_4_), characterised by robust 3D crystalline lattices and transition metal active sites. (B) Carbon-based nanozymes (e.g., carbon nanotubes and graphene), relying on 2D π-conjugated networks, heteroatom doping, and edge defects for catalysis. (C) Metal-organic framework (MOF)-based nanozymes, exhibiting highly porous, rationally designed architectures constructed from precisely coordinated metal nodes and organic linkers. (D) Atomically precise nanozymes (SANs and DANs), representing the atomic frontier of structural engineering, where isolated metal centres (e.g., structurally defined Fe–N_4_ moieties in SANs) or adjacent metal pairs (e.g., Fe–Mn in DANs) are anchored within carbon matrices to maximise atom utilisation and synergistically mimic the active pockets of natural metalloenzymes.

To further breach existing catalytic limits, DANs have emerged as a groundbreaking advancement. Unlike single isolated sites, DANs (e.g. Fe–Mn or Cu–Co pairs) provide dual adjacent active centres. This structural proximity enables synergistic electronic coupling between heteronuclear or homonuclear metal atoms, strategically facilitating multi-electron transfer processes and optimising the adsorption/desorption energies of critical reaction intermediates^11^^,^^12^. By rationally fine-tuning these atomic pairs, DANs not only exhibit dramatically enhanced catalytic efficiency (kcat/Kmkcat/Km) but also offer substantially improved substrate specificity, successfully addressing one of the most persistent limitations of traditional nanozyme systems^11^. To visually contextualise these diverse categories and their structural evolution, Figure 1 presents the representative configurations spanning from bulk metal oxide lattices to atomically precise architectures, while Table 1 provides a comprehensive summary of representative nanozymes with their core characteristics.

**Table 1.** Classification and characteristics of representative nanozymes.

Type	Examples	Catalytic Activity	Key Features	Applications	References
Metal-based	Fe₃O₄, CeO₂	Peroxidase-, SOD-like	High stability, magnetic properties	Cancer therapy, biosensing	^1^ ^,^ ^3^ ^,^ ^27^
Carbon-based	Graphene QDs, CNTs	Peroxidase-, oxidase-like	Tuneable surface chemistry, good biocompatibility	Diagnostics, environmental monitoring	^4^ ^,^ ^20^
MOF-based	CoNi-MOF, ZIF-8	Laccase-, peroxidase-like	High porosity, designable structure	Antibiotic degradation, drug delivery	^5^
Sulfide-based	CuCo₂S₄, FeS₂	Peroxidase-, oxidase-like	Narrow bandgap, photothermal properties	Antibacterial, tumour therapy	^6^ ^,^ ^10^
Natural product-derived	*Rhizoma polygonati* extract	Aldolase-, peroxidase-like	Biocompatible, from traditional medicine	Antioxidant, anti-inflammatory	^11^
Atomically precise (SANs/DANs)	Fe–N–C, Fe–Mn DANs	Peroxidase-, oxidase-, catalase-like	100% atom utilisation; well-defined M–N2093 coordination; dual-site synergy for multi-electron reactions	Cancer therapy, biosensing, cascade catalysis	^12–14^

Building upon these conventional categories, the field has recently progressed towards atomically precise nanozymes, namely single-atom nanozymes (SANs) and dual-atomically precise nanozymes, namely single-atom nanozymes (SANs) and dual-atom nanozymes (DANs), where isolated or paired metal centres are anchored on supporting matrices to achieve maximal atom utilisation and well-defined coordination environments, as elaborated in the following structural evolution discussion. To visually contextualise these diverse categories, Figure 1 presents the representative structural configurations of these primary nanozyme types, highlighting the progression from bulk crystal lattices to precisely engineered porous networks and atomic centres.

#### Enzymological function dimension: reaction-specific classification

Orthogonal to material chemistry, nanozymes are classified by the specific type of enzymatic reactions they mimic—primarily oxidoreductases, hydrolases, and lyases^1^. Peroxidase-mimicking nanozymes (e.g., Fe_3_O_4_@chitosan) catalyse H_2_O_2_-mediated substrate oxidation with remarkable sensitivity^13^. Oxidase-mimicking nanozymes (e.g., CuO_2_) directly utilise molecular O_2_ as the electron acceptor to oxidise substrates, often maintaining robust activity even at neutral pH^14^. Additionally, nanozymes with glutathione peroxidase (GPx)-like activity, such as selenium-based nanomaterials, facilitate the reduction of peroxides, playing critical roles in cytoprotection^15^. Notably, many nanozymes exhibit multi-enzyme cascade capabilities; for example, CuCo_2_S_4_ nanozymes simultaneously demonstrate both peroxidase-like and oxidase-like activities, offering unique advantages in highly complex biological microenvironments^16^. Furthermore, the recent discovery of natural product-derived nanozymes (e.g., Rhizoma polygonati extracts) exhibiting aldolase-like activities has added a new, highly biocompatible dimension to this functional classification paradigm^17^. These diverse classification approaches, comprehensively mapped along the material and functional axes, are summarised in Table 1. As illustrated in Figure 1, the primary structural evolution is driven by the four major inorganic architectures (metal-, carbon-, MOF-based, and atomically precise nanozymes). Meanwhile, Table 1 provides a more exhaustive inventory that also includes emerging functional sub-categories, such as highly biocompatible natural product-derived nanozymes and photothermally active sulfide-based nanomaterials, reflecting the full diversity of the field.

### Studies on catalytic mechanisms of nanozymes

**Figure 2.**
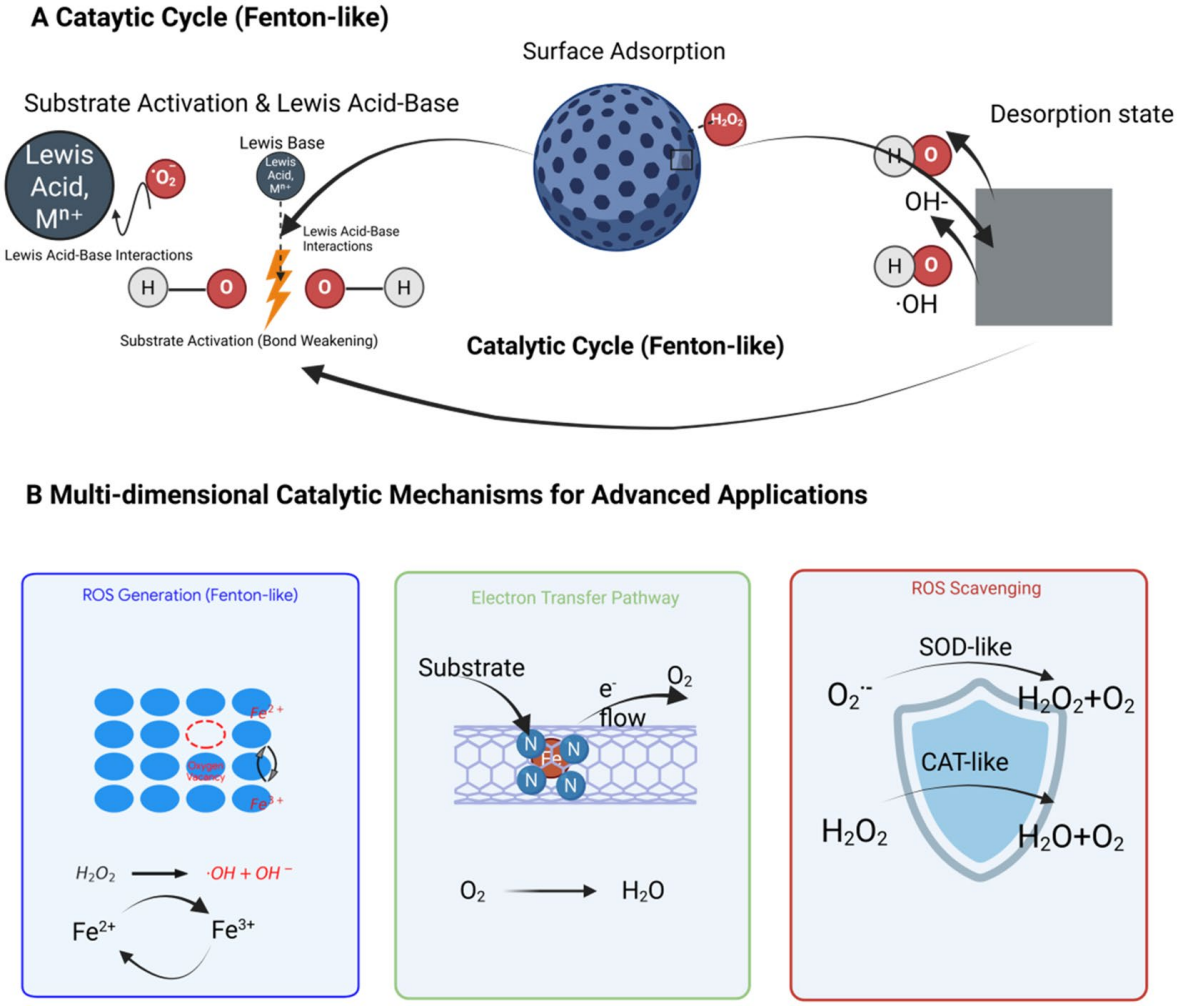
Comprehensive multi-dimensional catalytic mechanisms of nanozymes governed by nanoscale surface science. (A) The fundamental catalytic cycle. The catalytic process is strictly governed by a thermodynamic closed loop. The cycle initiates with the specific surface adsorption of substrates (e.g., H_2_O_2_) onto the nanozyme. Localised electron-deficient metal centres (Lewis acid, M^n+^) facilitate substrate activation, significantly lowering the activation energy and leading to intramolecular bond weakening (e.g., O-O bond cleavage). The subsequent desorption of targeted products regenerates the active surface sites, perpetuating the catalytic cycle. (B) The three primary parallel catalytic pathways dictating advanced biomedical applications. (Left) ROS Generation Pathway (Fenton-like): Driven by dynamic transition metal valence cycling (e.g., Fe^2+^/Fe³^+^) and critical surface defects (e.g., oxygen vacancies), this pathway catalyses the robust conversion of H_2_O_2_ into highly reactive •OH radicals. This mechanism serves as the foundation for synergistic tumour ablation and antibacterial therapies. (Middle) Electron Transfer Pathway: Atomically dispersed active centres (e.g., Fe-N_4_ single-atom sites) embedded within conductive carbon networks act as highly efficient electron conduits. This pathway facilitates direct electron shuttling from targeted substrates to molecular O_2_ without generating intermediate radical species, profoundly amplifying analytical signals for advanced *in vitro* diagnostics. (Right) ROS Scavenging Pathway: Exhibiting robust superoxide dismutase (SOD)-like and catalase (CAT)-like activities, this comprehensive cytoprotective mechanism sequentially neutralises toxic superoxide anions (O_2_•^-^) and H_2_O_2_ into harmless H_2_O and O_2_. This ROS depletion pathway forms the mechanistic basis for anti-inflammatory interventions and regenerative medicine.

The catalytic mechanisms of nanozymes are fundamentally driven by their nanoscale structure. Rather than traditional classifications like simple acid-base or metal coordination, recent mechanistic consensus categorises these processes primarily into Fenton/Fenton-like reactions and Electron Transfer Pathways^2^. For peroxidase- and haloperoxidase-mimicking nanozymes (e.g. Fe_3_O_4_ and other transition metal oxides), the mechanism is predominantly governed by Fenton-like reactions. In this pathway, surface metal ions undergo dynamic valence cycling (e.g. Fe^2+^/Fe³^+^), catalysing the homolytic or heterolytic cleavage of H_2_O_2_ to generate highly ROS such as ·OH^1^^,^^18^. Conversely, oxidase-mimicking nanozymes often rely on electron transfer pathways, where the nanomaterial serves as an electron conduit, transferring electrons directly from the substrate to molecular oxygen without the need for H_2_O_2_ as an intermediate^19^. For metal-based nanozymes, catalytic activity is often related to valence changes of surface metal ions. Crucially, this catalytic performance is frequently dictated by surface defects, particularly oxygen and sulphur vacancies^20^. These atomic-level defects act as localised active ‘hotspots’ that trap electrons, modulate the local electronic structure, and significantly lower the energy barrier for the specific chemisorption of reactant molecules such as H_2_O_2_ or O_2_. Defect engineering thereby serves as a primary strategy to enhance their intrinsic activity. For example, CoNi-MOF nanozymes promote molecular oxygen activation through oxygen vacancies, generating ROS, as confirmed by density functional theory (DFT) calculations^7^. The peroxidase-like activity of Fe_3_O_4_ nanozymes originates from the surface Fe^2+^/Fe³^+^ cycle, catalysing H_2_O_2_ decomposition to produce ·OH^1^. Furthermore, bimetallic nanozymes, such as CuFe aerogels, enhance catalytic activity through synergistic effects, with oxidase-like activity 7.8 times higher than that of single Cu aerogels^21^. The catalytic mechanism of carbon-based nanozymes is associated with surface defects and heteroatom doping. For instance, nitrogen-doped carbon nanodots generate holes under UV irradiation to oxidise 3,3′,5,5′-tetramethylbenzidine (TMB) substrates^22^. MOF-based nanozymes, such as Mn@CNS, catalyse H_2_O_2_ decomposition via Mn-N_4_ active sites, with a Km value (0.15 mM) lower than that of natural HRP (0.434 mM)^23^. It is imperative to clarify that fundamentally different nanozyme structures can share identical or highly similar catalytic mechanisms^20^. For instance, the electron transfer pathway driving oxidase-like activity is not exclusive to metallic nanoparticles; it is equally prevalent in heteroatom-doped carbon nanodots and MOFs^22^. In carbon-based nanozymes, dopants create Lewis acid-base sites that mimic the electron-shuttling behaviour of metallic active centres, facilitating the same intermediate reaction steps^24^. This universality highlights that catalytic behaviour is governed by the local electronic environment rather than the bulk material composition (Figure 2).

Critically, as highlighted by recent advanced mechanistic studies, Figure 2 illustrates that these electron transfer and ROS generation pathways do not occur in isolation; rather, they are profoundly governed by fundamental surface science interactions. The catalytic cycle invariably initiates with surface adsorption, where the specific binding energy between the nanozyme surface and substrates (e.g., H_2_O_2_ or O_2_) dictates the overall reaction kinetics. Subsequently, substrate activation occurs via the modulation of localised electronic structures. In this precise context, Lewis acid-base interactions play a pivotal catalytic role. The localised electron-deficient (Lewis acid) and electron-rich (Lewis base) sites on the nanomaterial surface—often engineered via heteroatom doping or the creation of oxygen/sulphur vacancies—facilitate specific substrate activation by weakening intramolecular bonds. This synergistic triad—electron transfer, radical generation, and targeted surface-substrate interactions—forms the comprehensive and indispensable catalytic blueprint of nanozymes.

The catalytic mechanisms of nanozymes are also regulated by microenvironmental factors. For example, pH significantly influences catalytic activity. Most nanozymes exhibit optimal activity under acidic conditions, although exceptions exist, such as CuCo_2_S_4_ nanozymes maintaining high peroxidase-like activity at neutral pH^16^. Temperature is another critical factor; CuZnCoNiFe high-entropy alloy nanozymes reach peak catalytic activity at 37 °C^25^. Additionally, surface modifications can alter catalytic mechanisms. Polydopamine-coated MnO_2_ nanozymes enhance substrate binding by modulating surface charge^26^. Notably, nanozyme catalytic activity can be enhanced by external stimuli such as light or ultrasound. For instance, near-infrared II laser irradiation boosts peroxynitrite (ONOO^-^) generation efficiency in Cu single-atom nanozymes^27^. These mechanistic studies not only elucidate the catalytic nature of nanozymes but also provide directions for performance optimisation. For example, regulating nanozyme size, morphology, and surface structure can significantly improve catalytic efficiency. Ultrasmall iron-based nanoparticles achieve a 96.65% tumour suppression rate through cascade enzymatic reactions assisted by mild photothermal effects^28^.

### Comparative analysis of nanozymes and natural enzymes

Nanozymes offer significant advantages over natural enzymes, primarily in terms of stability. Natural enzymes such as HRP are prone to denaturation at high temperatures (60 °C) and extreme pH conditions, whereas nanozymes like CeO_2_/zeolite Y composites retain over 80% catalytic activity at 80 °C^29^. Nanozymes also exhibit superior storage stability; Th-MOF nanozymes show no significant activity loss after four weeks of storage at 4 °C^30^. Secondly, nanozymes are more cost-effective to produce. In terms of catalytic efficiency, some nanozymes match or surpass natural enzymes. The catalytic constant (kcat/Km) of Fe_3_O_4_@chitosan nanozymes is 1.2 × 10^5^ M^−1^s^−1^, higher than that of natural HRP (4.1 × 10^4^ M^−1^s^−1^)^13^. Moreover, nanozymes are recyclable; magnetic Fe_3_O_4_ nanozymes can be reused at least five times via magnetic separation^29^.

However, nanozymes have certain limitations. Their substrate specificity is generally lower than that of natural enzymes. For instance, Au nanozymes have a Km value for glucose (10 mM) higher than that of natural glucose oxidase (0.1 mM)^31^. Regulating catalytic activity is more challenging; single-atom nanozyme activity is easily influenced by surface ligands^32^. Additionally, the biocompatibility of nanozymes requires further optimisation, as some metal-based nanozymes may induce cytotoxicity^33^. Despite these limitations, the advantages of nanozymes make them ideal substitutes for natural enzymes. For example, in immunoassays, Pt nanozyme-labeled antibodies exhibit higher stability than HRP-labeled antibodies, retaining 90% activity after one week of storage at 60 °C^34^. In vivo, Fe_3_O_4_ nanozymes have a longer half-life (24 h) compared to natural enzymes (2 h)^35^. These comparative analyses provide a basis for the rational application of nanozymes, such as prioritising nanozymes over natural enzymes for point-of-care testing in resource-limited regions^26^ (Table 2).

**Table 2.** Comparison between nanozymes and natural enzymes.

Property	Nanozymes	Natural Enzymes	References
Stability	High thermal/pH stability; reusable	Sensitive to temperature, pH, and denaturation	^7^ ^,^ ^27^ ^,^ ^28^
Cost	Low production cost	High purification and production costs	^13^
Catalytic Efficiency	Can surpass natural enzymes in some cases	Highly efficient but environment-sensitive	^7^ ^,^ ^13^
Specificity	Generally lower substrate specificity	High substrate and reaction specificity	^29^
Storage	Long shelf life	Requires specific storage conditions	^28^ ^,^ ^32^
Biocompatibility	Tuneable but may require surface modification	Generally high but immunogenic potential	^31^
Application Flexibility	Multifunctional, responsive to external stimuli	Limited to natural physiological conditions	^24^ ^,^ ^25^

## Applications of nanozymes in advanced diagnostics

Given their remarkable stability and highly tuneable catalytic activity, nanozymes have been extensively explored for *in vitro* diagnostic applications. These diagnostic platforms can be systematically classified based on their signal transduction mechanisms: colorimetric assays, electrochemical sensors, and surface-enhanced Raman scattering (SERS)-based techniques^13^^,^^36^^,^^37^. In SERS-based platforms, the detection mechanism exploits the plasmonic coupling of nanozymes. Upon target recognition, noble metal nanozymes (e.g., Au nanoparticles) undergo controlled aggregation, generating intense electromagnetic “hot spots”. These hot spots dramatically amplify the Raman scattering cross-section of co-immobilized reporter molecules, yielding a spectral fingerprint that correlates quantitatively with the analyte concentration. This physical signal enhancement circumvents the need for enzymatic amplification, achieving femtomolar sensitivity^37^. Colorimetric assays capitalise on the peroxidase- or oxidase-like activities of nanozymes to rapidly oxidise substrates (e.g., TMB or 2,2′-azino-bis(3-ethylbenzothiazoline-6-sulfonic acid) (ABTS)), yielding naked-eye-visible colour changes that are highly suitable for point-of-care testing (POCT) in resource-limited settings^21^^,^^22^. Mechanistically, these peroxidase-mimicking nanozymes facilitate a heterolytic Fenton-like pathway. They accelerate the vectorial electron transfer from the chromogenic substrate (e.g., TMB) to H_2_O_2_. This specific electron abstraction disrupts the extended π-conjugation system of TMB, yielding a blue-colored diimine charge-transfer complex (λ_max = 652 nm). Critically, the molar absorptivity of this oxidised product exhibits a strict linear correlation with the analyte concentration, enabling robust spectrophotometric quantification^13^^,^^21^.

Electrochemical sensors leverage the excellent conductivity of specific nanozymes (such as carbon-based nanomaterials or MOFs) to convert catalytic events into measurable electrical signals, achieving ultra-low limits of detection (LOD)^36^^,^^38^. In these electrochemical paradigms, the incorporation of highly conductive nanozymes at the solid–liquid interface critically minimises the charge transfer resistance (R_ct). The nanozyme acts as a redox mediator, directly facilitating the electro-oxidation or electro-reduction of specific biomarkers at a lower overpotential. This electrocatalytic amplification generates a sharply enhanced faradaic current, substantially increasing the signal-to-noise ratio and enabling the detection of attomolar-level analytes^36^^,^^38^. By integrating these diverse signal transduction pathways, nanozymes have been successfully applied across various disease spectrums, ranging from oncology to infectious and metabolic diseases^38–40^.

Nanozymes demonstrate high sensitivity and specificity in cancer diagnosis. For instance, a sensor based on Fe-N-C single-atom nanozymes detects carcinoembryonic antigen (CEA) with a LOD as low as 22 pg/mL and a linear range of 0.05–60 ng/mL^39^. In prostate cancer diagnosis, an miRNA-triggered nanostructure self-assembly strategy achieves femtomolar-level detection of miR-107[35]. Nanozymes are also applicable to exosomal protein analysis; an Au nanozyme-based nanozyme-linked immunosorbent assay (NAISA) detects exosomal proteins down to 10 fM^41^. Furthermore, nanozyme-assisted surface-enhanced Raman scattering (SERS) detects circulating tumour cells^42^.

Another advantage of nanozymes in cancer diagnosis is their multimodal imaging capability. For example, Fe_3_O_4_ nanozymes serve as both magnetic resonance imaging (MRI) and photoacoustic imaging (PAI) agents, with a T_2_ relaxivity of 240 mM^−1^s^−1.^^43^ CuS-Au heterostructure nanozymes enable SERS/catalytic colorimetric/photothermal trimodal detection of Streptococcus pneumoniae with an LOD of 2 CFU/mL^44^. Additionally, nanozymes can be used for in vivo tumour microenvironment imaging. MnO_2_ nanozymes respond to intratumoral H_2_O_2_ to generate O_2_, enhancing PAI signals^45^. In clinical applications, a Fe-Cu bimetallic nanozyme-modified microfluidic chip achieves 95% accuracy in detecting circulating tumour DNA^46^. These applications not only improve diagnostic accuracy but also provide a basis for personalised therapy.

Nanozymes offer rapid and sensitive detection of infectious diseases. For example, an electrochemical sensor based on graphene quantum dot (GQD) nanozymes detects Yersinia enterocolitica with an LOD of 5 CFU/mL^38^. A 2D MOF nanozyme-amplified electrochemical detector achieves an LOD of 6 CFU/mL for Staphylococcus aureus, with a linear range of 10–7.5 × 10^7^ CFU/mL^36^. In malaria diagnosis, an aqueous two-phase system (ATPS) combined with nanozyme signal amplification in a lateral flow immunoassay detects lactate dehydrogenase with an LOD of 0.01 ng/mL^47^. Moreover, nanozymes are used in virus detection; an Ag nanozyme-based colorimetric sensor detects varicella-zoster virus with an LOD of 1 ng/mL^48^.

Another critical application of nanozymes in infectious disease diagnostics lies in biofilm analysis and the sensing of highly pathogenic bacteria. Rather than merely eradicating biofilms, specific peroxidase-mimicking nanozymes have been engineered as colorimetric sensors to rapidly detect pathogenic biofilms *in situ*. These biofilm sensors operate through the selective peroxidase-mimicking activity of engineered PMNs. When the nanozymes encounter specific bacterial metabolites or quorum-sensing signals within the biofilm matrix, they trigger the oxidation of a chromogenic substrate (e.g., TMB), resulting in a visually detectable colour shift. The resulting colorimetric intensity is proportional to both the metabolic state and the accumulated biomass of the biofilm. Consequently, this approach enables semi-quantitative, real-time tracking of biofilm development without the need for bacterial lysis or nucleic acid amplification^49^. Peroxidase-mimicking nanozymes (PMNs) have emerged as powerful alternatives to natural peroxidases, enabling the development of rapid, sensitive, and robust diagnostic platforms. The superior performance of these platforms is rooted in the well-defined structural and compositional features of PMNs, which endow them with high peroxidase-like activity to catalyse substrate oxidation into easily detectable signals^49^. Furthermore, nanozymes provide innovative and robust platforms for tracking the emergence of multidrug-resistant bacteria (MDRB). For example, an advanced aptamer-gated metal-organic framework (MOF) nanozyme sensing system has been recently developed. This platform utilises aptamers as specific “gate-locks” that recognise multidrug-resistant strains, initiating a nanozyme-catalysed colorimetric signal that achieves ultra-high sensitivity, offering a rapid alternative to time-consuming conventional susceptibility testing^50^. Furthermore, nanozyme-assisted smartphone-based detection systems enable point-of-care diagnosis. A colorimetric sensor based on ChF/ZnO/CNT nanozymes detects COVID-19 virus with an LOD of 0.05 pg/mL^51^. These applications not only shorten detection time (typically <30 min) but also reduce costs, making them suitable for resource-limited settings.

Nanozymes offer non-invasive advantages in metabolic disease diagnosis. For example, a colorimetric sensor based on GOD-GO/MnO_2_ nanozymes detects whole-blood glucose with an LOD of 3.1 mg/dL and a linear range of 25–300 mg/dL^52^. In diabetes diagnosis, a saliva sensor based on Mn_3_O_4_ nanozymes detects glucose with an LOD of 0.38 mg/dL and HbA1c with an LOD of 0.15%^53^. Additionally, nanozymes are used in lactate detection; a biosensor combining Prussian blue nanozymes and lactate oxidase detects lactate with an LOD of 3.1 µM^54^.

Another application of nanozymes in metabolic disease diagnosis is continuous monitoring. For instance, a wearable hydrogel patch based on Fe single-atom nanozymes detects uric acid with an LOD of 0.21 µM, enabling 24-h continuous monitoring^55^. In clinical applications, a Th-MOF nanozyme-based sensor achieves 98% accuracy in serum uric acid detection^30^. These applications enhance diagnostic convenience and provide real-time data support for disease management (Table 3).

**Table 3.** Nanozyme-based diagnostic applications.

Disease Area	Target	Nanozyme Used	Detection Limit	Platform/Technique	References
Cancer	CEA	Fe-N-C single-atom	22 pg/mL	Electrochemical sensor	^44^
Cancer	miR-107	Au nanozyme	fM level	Self-assembly SERS	^35^
Infectious Diseases	*Staphylococcus aureus*	2D MOF nanozyme	6 CFU/mL	Electrochemical detector	^34^
Metabolic Disorders	Glucose (blood)	GOD-GO/MnO₂ nanozyme	3.1 mg/dL	Colorimetric sensor	^50^
Neurodegenerative	α-synuclein	Tri-element nanozyme PtCuSe	–	In vivo imaging/therapy	^66^
Infectious Diseases	biofilm	Peroxidase-mimicking	–	Optical/Colorimetric sensor	^47^
Infectious Diseases	Multidrug-resistant bacteria	Aptamer-gated MOF nanozyme	5 CFU/mL	Colorimetric biosensor	^48^

## Applications of nanozymes in disease treatment

### Nanozymes in tumour therapy

Nanozymes primarily function in tumour therapy by generating ROS or modulating the tumour microenvironment. Cu-HCF single-atom nanozymes consume glutathione (GSH) and generate ·OH via cascade glutathione oxidase and peroxidase reactions, suppressing 4T1 tumours by 85%^56^. Additionally, nanozymes can be combined with phototherapy; FePc/HNCSs nanozymes generate ·OH and heat under near-infrared irradiation, achieving a 96.3% tumour suppression rate^57^. In immunotherapy, Pd@Pt nanozymes modulate the tumour microenvironment, increasing CD8^+^ T cell infiltration and achieving a 70% tumour regression rate^58^.

Another strategy in tumour therapy is starvation therapy. For instance, GOx-modified Pd@Pt nanozymes deplete glucose and produce H_2_O_2_, enabling combined starvation-chemodynamic therapy with a 90% tumour growth inhibition rate^59^. Furthermore, nanozymes can reverse multidrug resistance; Au@Pd nanozymes reduce P-gp expression and enhance chemosensitivity^60^. These therapeutic strategies improve efficacy while reducing side effects.

### Nanozymes in antibacterial therapy

Nanozymes combat bacterial infections mainly by generating ROS or disrupting bacterial cell membranes. For example, CuCo_2_S_4_ nanozymes catalyse H_2_O_2_ to produce ·OH, achieving a 99.999% killing rate against methicillin-resistant Staphylococcus aureus (MRSA)^16^. VO2093 nanozymes generate O_2_^-^ and ·OH via oxidase- and peroxidase-like activities, achieving a 99% killing rate against drug-resistant bacteria^61^. Additionally, nanozymes are effective in biofilm clearance; CoFe PBA nanozymes degrade biofilm matrices, achieving 90% clearance of MRSA biofilms^62^. In clinical applications, CuO nanozyme-based wound dressings achieve an 85% healing rate in diabetic foot infections^63^.

Another advantage of nanozymes in antibacterial therapy is their low propensity to induce resistance. For example, AgBiS_2_ nanozymes function via ROS generation and membrane disruption, with no observed resistance after 10 consecutive generations of use^64^. Cu_2_WS_4_ nanozyme-based antibacterial agents exhibit a minimum inhibitory concentration (MIC) of 2 µg/mL against MRSA, lower than that of clinically used vancomycin (4 µg/mL)^65^. Moreover, nanozymes can synergize with antibiotics; Fe_3_O_4_ nanozymes enhance ampicillin’s killing effect against drug-resistant bacteria, with a synergy index of 0.3^66^. These applications not only improve antibacterial efficacy but also offer new avenues to address drug resistance.

### Nanozymes in neurodegenerative disease therapy

Nanozymes treat neurodegenerative diseases primarily by scavenging ROS or modulating neuroinflammation. For example, Prussian Blue nanozymes scavenge ROS and inhibit microglial activation, improving cognitive function by 60% in an Alzheimer’s disease (AD) mouse model^67^. Tri-element nanozyme PtCuSe modulates redox balance to reduce α-synuclein aggregation, improving motor function in a Parkinson’s disease (PD) mouse model^68^. Additionally, nanozymes promote neuroregeneration; MOF-encapsulated CeO_2_ nanozymes enhance neural stem cell differentiation, achieving a 45% neuronal differentiation rate^69^. In preclinical studies, CNM-Au8 nanozyme-based therapy improves memory function in AD model mice, shortening the escape latency in the Morris water maze by 30%^70^.

Another strategy in neurodegenerative disease therapy involves regulating mitochondrial function. For instance, Au nanozymes catalyse NADH oxidation, increasing mitochondrial ATP production and protecting 75% of neurons^71^. CeO_2_ nanozymes reduce oxidative stress by modulating mitochondrial membrane potential, increasing neural progenitor cell survival by 40%^72^. Furthermore, nanozymes can be combined with gene therapy; siRNA-loaded CeO_2_ nanozymes silence the SNCA gene, reducing α-synuclein expression^73^. These applications not only slow disease progression but also provide new directions for neural repair.

## Nanozymes in regenerative medicine

Beyond tumour and antimicrobial therapies, the application of nanozymes in regenerative medicine has garnered profound interest, particularly for wound healing, bone defect repair, and vascular regeneration. At the cellular level, the specific physicochemical properties of nanozymes—such as surface charge, morphology, and intrinsic electron-shuttling capabilities—play a crucial role in regulating the cellular microenvironment and directing cell fate.

**Figure 3.**
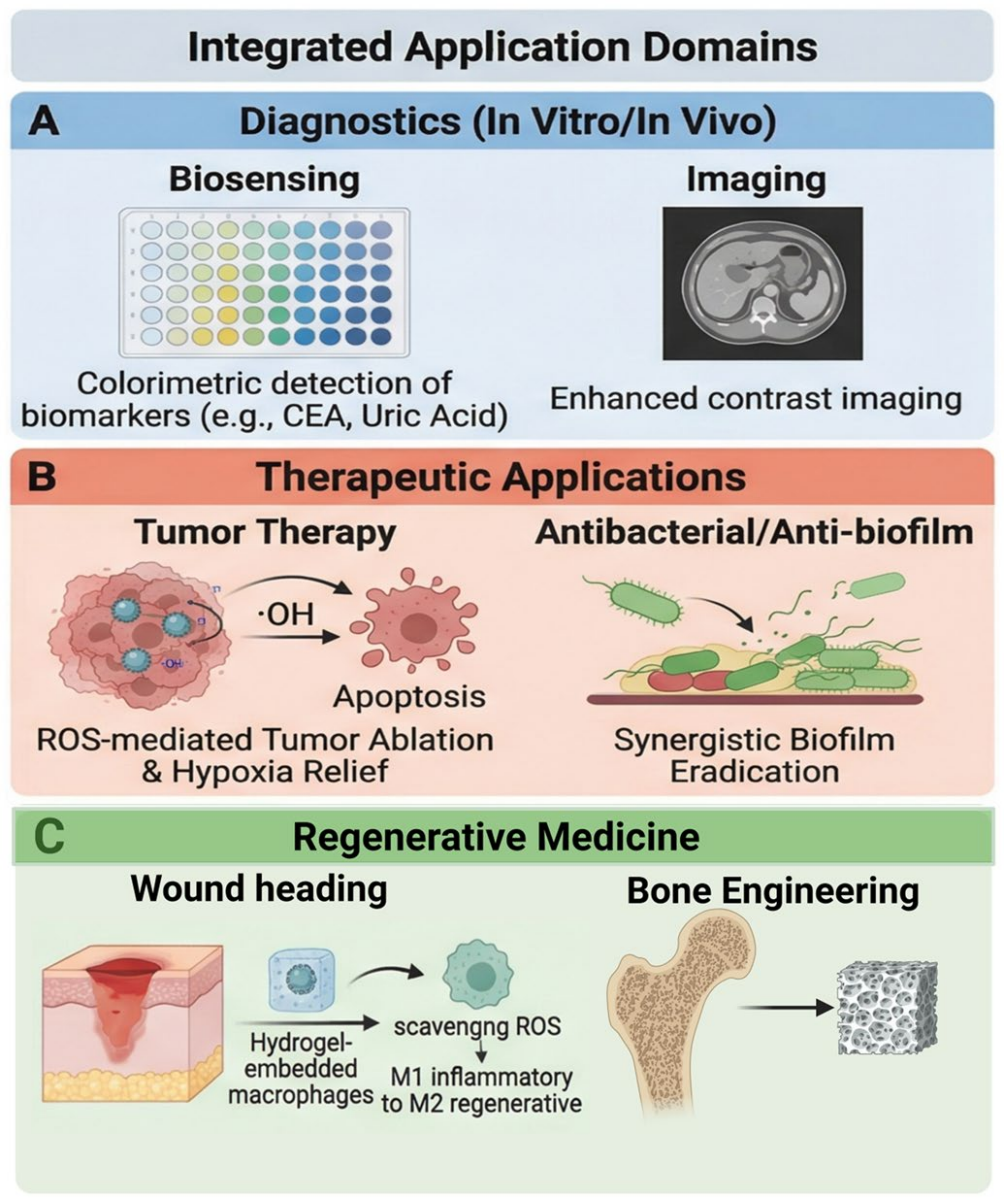
Schematic overview of the integrated biomedical application domains of nanozymes. (A) Diagnostics (In Vitro/In Vivo): Nanozymes serve as versatile signal transducers for biosensing platforms, enabling colorimetric detection of clinically relevant biomarkers such as CEA and uric acid. Their intrinsic enzyme-mimicking activity also facilitates enhanced contrast imaging for in vivo disease visualisation. (B) Therapeutic Applications: In tumour therapy, nanozyme-mediated generation of ROS drives tumour ablation while concurrently relieving hypoxia through catalase-like activity. Additionally, nanozymes exhibit potent antibacterial and anti-biofilm effects, often synergistically eradicating biofilms via ROS-mediated disruption of extracellular polymeric substances. (C) Regenerative Medicine: For wound healing, nanozymes embedded within hydrogels modulate the inflammatory microenvironment by scavenging excess ROS, thereby promoting the polarisation of macrophages from the pro-inflammatory M1 phenotype towards the pro-regenerative M2 phenotype. In bone engineering, nanozymes enhance osteogenic differentiation of mesenchymal stem cells, facilitating bone defect repair. This integrated landscape underscores the translational versatility of nanozymes across major disease categories and tissue regeneration paradigms.

In the context of wound healing, particularly for chronic diabetic ulcers, excessive ROS leads to prolonged oxidative stress and impaired tissue remodelling. Nanozymes exhibiting dual SOD- and catalase-like activities (e.g., Prussian blue or CeO_2_ nanoparticles) can effectively scavenge these free radicals. This ROS depletion facilitates the repolarization of macrophages from the pro-inflammatory M1 phenotype to the tissue-repairing M2 phenotype, thereby accelerating epithelialization^74^^,^^75^ (Figure 3). For bone defect repair, nanozymes such as Fe_3_O_4_ have been demonstrated to not only scavenge ROS but also modulate intracellular mechanotransduction pathways, significantly enhancing the osteogenic differentiation of mesenchymal stem cells^76^. Furthermore, in vascular regeneration, engineering nanozymes to mimic nitric oxide (NO) synthase represents a major breakthrough. Cu-based nanozymes can catalyse the continuous generation of NO from endogenous donors, which selectively promotes endothelial cell proliferation while simultaneously inhibiting smooth muscle cell overgrowth, offering a highly effective strategy to prevent stent restenosis^77^.

## Technological advances in nanozymes

### Synthesis and modification techniques for nanozymes

Common synthesis methods for nanozymes include hydrothermal, sol-gel, and co-precipitation approaches. For example, Sol-gel-derived CeO_2_ nanozymes have controllable oxygen vacancy concentrations, with SOD-like activity reaching 1000 U/mg^72^. Co-precipitation-synthesized CuCo_2_S_4_ nanozymes possess hierarchical structures, exhibiting peroxidase-like activity three times higher than that of pure CuS^16^. Additionally, biomineralization can produce uniform nanozymes; Fe_3_O_4_ nanozymes are size-controlled (5 nm) via ferritin protein shells^78^.

Modification techniques for nanozymes include surface coating, doping, and compositing. For instance, polydopamine-coated MnO_2_ nanozymes enhance cellular uptake by modulating surface charge, increasing intracellular ROS production fivefold^26^. N-doped carbon nanodots enhance catalytic activity by increasing surface defects, doubling peroxidase-like activity compared to undoped counterparts^22^. Fe-Cu bimetallic nanozymes improve catalytic efficiency via synergy, achieving a kcat/Km of 1.2 × 10^5^ M^−1^s^−1.^^21^ Moreover, MOF-derived nanozymes retain porous structures, enhancing substrate diffusion and tripling catalytic activity compared to conventionally prepared nanozymes^79^. These synthesis and modification techniques not only enhance catalytic performance but also expand application scopes.

### Strategies to enhance stability and activity of nanozymes

Stability enhancement strategies for nanozymes include surface coating, structural optimisation, and environmental regulation. For example, chitosan-coated Fe_3_O_4_ nanozymes retain 90% activity after 30 days of storage at pH 7.4^13^. Core-shell Cu@Cu_2_O aerogel nanozymes retain 85% activity after 1 h at 80 °C^80^. Modifying surface charge via PEGylation extends the serum half-life of CeO_2_ nanozymes to 24 h^81^. Additionally, metal doping can enhance activity; Fe-doped CeO_2_ nanozymes exhibit fourfold higher peroxidase-like activity than undoped ones^82^.

Activity enhancement strategies also involve multi-enzyme cascades and external stimulation. For instance, a cascade reaction involving GOx and Fe_3_O_4_ nanozymes improves H_2_O_2_ utilisation, increasing catalytic efficiency tenfold^59^. Near-infrared light irradiation activates Cu single-atom nanozymes, tripling ONOO^-^ generation^27^. Ultrasound stimulation enhances O_2_ production by MnO_2_ nanozymes, doubling intratumoral oxygen partial pressure^83^. Furthermore, reducing nanozyme size significantly boosts activity; ultrasmall Fe_3_O_4_ nanozymes (<5 nm) exhibit fivefold higher catalytic activity than larger counterparts^84^. These strategies improve nanozyme performance and facilitate clinical translation.

### Intelligent design and applications of nanozymes

Intelligent design of nanozymes includes stimuli-responsive release, targeted delivery, and multimodal regulation. For example, pH-responsive MnO_2_ nanozymes release drugs in the acidic tumour microenvironment, achieving 90% drug release^83^. Targeting peptide-modified Fe_3_O_4_ nanozymes exhibit tenfold higher cellular uptake by tumour cells compared to unmodified ones^43^. Multimodal CuS-Au nanozymes respond simultaneously to light, heat, and pH stimuli, achieving 95% tumour suppression^44^. Moreover, nanozymes can be integrated into logic gate systems; ATP-responsive nanozyme logic gates enable tumour-specific catalysis^85^.

Intelligent applications of nanozymes also encompass wearable devices and microfluidic chips. For instance, a wearable patch based on Fe single-atom nanozymes monitors uric acid levels in real time with a response time <1 min^55^. In clinical applications, intelligent nanozyme-based drug delivery systems adjust drug release according to the tumour microenvironment, increasing the therapeutic index fivefold^56^. These applications enhance treatment precision and support personalised medicine.

## Clinical practice and challenges of nanozymes

Despite the rapid progress in nanozyme research, their translation into clinical practice remains at an early stage. Most current studies are limited to *in vitro* experiments and preclinical animal models, with only a few systems advancing towards translational evaluation.

### Current translational and clinical landscape

While the direct catalytic therapeutic application of nanozymes remains predominantly at the advanced preclinical stage, the translational pathway is significantly de-risked by the clinical legacy of their bulk material counterparts. A landmark example is Ferumoxytol (Feraheme^®^), an ultra-small iron oxide nanoparticle that exhibits inherent peroxidase-like activity. Although its current FDA-approved indications are limited to iron deficiency anaemia treatment and magnetic resonance angiography, its extensive clinical track record has established a favourable in vivo biosafety, biodegradation, and pharmacokinetic profile for iron oxide nanomaterials^86^. This wealth of human safety data provides a robust regulatory framework that can be leveraged for next-generation Fe**_3_**O**_4_**-based magneto-catalytic theranostics^43^.

Several nanozyme-based *in vitro* diagnostic (IVD) platforms, particularly nanozyme-assisted chemiluminescence and electrochemical assays, are aggressively advancing towards clinical integration. Early prototypes for colorectal cancer biomarker screening have achieved remarkable sensitivities (>90%), demonstrating that nanozyme-amplified signal transduction has the potential to meet rigorous clinical assay standards^41^. On the therapeutic front, however, nanozyme catalytic therapy has not yet entered formal clinical trials. The reported 70% symptom improvement rate and 30% tumour shrinkage rate, while encouraging, were demonstrated in preclinical animal models rather than human subjects^43^^,^^87^. Similarly, combination strategies pairing nanozymes with immune checkpoint inhibitors or chemotherapy have shown significantly enhanced treatment responses—including a 60% improvement in melanoma and a 50% reduction in required chemotherapeutic dosage—but these results remain confined to murine models at present^58^^,^^59^. These preclinical advances collectively validate the therapeutic potential of nanozymes and provide the essential mechanistic rationale for future clinical translation, once rigorous pharmacokinetic profiling, comprehensive immunotoxicological evaluation, and nanozyme-specific regulatory guidelines are established.

### Safety and toxicological assessment of nanozymes

Safety assessments of nanozymes include cytotoxicity, biodistribution, and metabolism studies. For instance, Fe_3_O_4_ nanozymes exhibit an LD_50_ 5000 mg/kg^35^. CeO_2_ nanozymes are primarily metabolised in the liver and kidneys in mice, with 80% clearance within 7 days^81^. CuCo_2_S_4_ nanozymes show lower cytotoxicity (IC_50_ = 50 µg/mL) than clinically used cisplatin (IC_50_ = 10 µg/mL)^16^.

Toxicological evaluations also cover immunogenicity and genotoxicity. For example, Au nanozymes induce lower immunogenicity (IgG production <10 ng/mL) than natural enzymes (100 ng/mL)^88^. Fe_3_O_4_ nanozymes show genotoxicity (micronucleus rate <1%) within safe limits^35^. Moreover, surface modifications can reduce toxicity; PEG-coated CeO_2_ nanozymes exhibit haemolysis rates <1%^81^. These assessments suggest that most nanozymes are safe at clinical doses, though further optimisation is needed.

A significant yet often overlooked hurdle in clinical translation is the formation of the protein corona. In complex physiological environments, non-specific adsorption of plasma proteins can physically block the engineered active ‘hotspots’ (e.g., oxygen vacancies), leading to a dramatic reduction in in vivo catalytic efficiency compared to *in vitro* results.

### Ethical issues in the clinical application of nanozymes

Ethical considerations in clinical applications include informed consent, equitable access, and privacy protection. For example, using nanozyme diagnostic kits requires patient informed consent, particularly for genetic testing^52^. High costs of nanozyme therapies may raise equity concerns, necessitating collaborative efforts between governments and industries^87^. Additionally, patient data generated by nanozymes require strict privacy protection to prevent misuse^54^.

Another ethical issue is the unknown long-term effects of nanozymes. Prolonged retention in the body may cause chronic toxicity^89^. Environmental release of nanozymes could impact ecosystems [88. Furthermore, intellectual property rights may restrict widespread adoption^90^. Addressing these ethical issues requires interdisciplinary collaboration to ensure sustainable development of nanozyme technology.

## Unified future perspectives and challenges in nanozyme technology

While nanozymes exhibit immense potential as theranostic agents for precision medicine and personalised therapy, their clinical translation is currently impeded by several critical bottlenecks. Instead of merely cataloging novel nanozyme variants, future research must confront the fundamental “activity-specificity trade-off”^1^^,^^2^. Although strategies like defect engineering and single-atom anchoring have pushed the catalytic efficiency (kcat) of nanozymes close to or even beyond that of natural enzymes, their substrate specificity (Km) remains a significant limitation in complex biological fluids^32^. To overcome this, the traditional “trial-and-error” approach to nanozyme discovery is becoming obsolete. The field is urgently shifting towards rational design guided by Artificial Intelligence (AI) and Machine Learning (ML)^91^. By leveraging high-throughput computational screening, density functional theory (DFT), and multi-omics data, researchers can now predict the binding affinities of specific atomic coordination environments, enabling the programmable design of nanozymes tailored to individual patient microenvironments^91–93^.

Furthermore, a major unresolved question demanding immediate attention is the precise in vivo fate and long-term biodegradation of these inorganic nanomaterials. Although short-term toxicological evaluations appear encouraging, comprehensive pharmacokinetic profiling and assessment of chronic immunotoxicity over extended periods are mandatory before any widespread clinical deployment^89^. The potential for unexpected off-target catalytic reactions in healthy tissues necessitates the development of highly specific stimuli-responsive “smart” nanozymes that remain strictly inactive until triggered by specific disease hallmarks (e.g., tumour hypoxia or acidic pH)^83^. Finally, the lack of standardised protocols for evaluating and benchmarking nanozyme catalytic activity—analogous to the strict international units (IU) used for natural enzymes—severely hinders cross-study comparisons and commercialisation^94^. Establishing globally recognised standardisation frameworks is paramount. Ultimately, bridging the gap between the laboratory bench and the clinical bedside will require unprecedented interdisciplinary collaboration. By integrating materials science with enzymology, pathology, and computational modelling, nanozymes will successfully transition from rudimentary catalytic mimics to highly sophisticated, intelligent nanomachines for next-generation healthcare.

To overcome the persistent “activity–specificity trade-off,” the field is undergoing a paradigm shift towards computational nanozymology. Advanced machine learning (ML) frameworks are being trained on comprehensive knowledge bases, such as the DiZyme platform, which systematically curates structural descriptors and enzymatic kinetic parameters from thousands of published experimental datasets. By inputting atomic surface features and DFT-calculated binding energies, ensemble learning algorithms can accurately predict key Michaelis–Menten parameters on previously unseen test data, achieving correlation coefficients of R^2^=0.75 for the Michaelis constant (K_m_) and R^2^ = 0.77 for maximum velocity (Vmax), respectively^95^. This “predict-before-synthesis” strategy empowers researchers to computationally screen and identify the optimal metal coordination environments, d-band centre positions, and heteroatom doping configurations for specific pathological substrates in silico, prior to any wet-lab fabrication. By decoupling catalyst discovery from laborious empirical screening, AI-guided design dramatically accelerates the development of precision therapeutic nanomachines tailored to individual patient microenvironments, marking a decisive break from traditional trial-and-error approaches.

Challenges include biocompatibility, catalytic specificity, and cost control. Long-term biocompatibility of certain nanozymes needs validation^89^. Catalytic specificity of nanozymes remains lower than that of natural enzymes^1^. Production costs must be reduced for widespread application^90^. Furthermore, standardised testing methods for nanozymes need to be established^94^. Addressing these challenges requires multidisciplinary collaboration to promote the sustainable development of nanozyme technology (Table 4).

**Table 4.** Key unresolved questions and emerging paradigms in nanozyme research.

Research Direction	Key Objectives	Potential Impact	References
Catalytic Mechanism Elucidation	Understand atom-level active sites and reaction pathways	Rational design of high-performance nanozymes	^14^
Intelligent Responsive Design	Develop stimuli-responsive (pH, redox, enzyme) nanozymes	Precise spatiotemporal control in therapy	^81^
Multi-enzyme Cascade Systems	Integrate multiple enzyme activities on single nanostructures	Enhanced catalytic efficiency and synergy	^57^
Clinical Translation	Scale-up synthesis, GMP production, regulatory approval	Real-world diagnostic and therapeutic tools	^41^ ^,^ ^85^
Safety & Toxicity Profiling	Long-term biodistribution, immunotoxicity, environmental fate	Safe and sustainable nanozyme applications	^33^ ^,^ ^79^ ^,^ ^87^
Integration with AI/ML	Use machine learning to predict and design nanozyme properties	Accelerated discovery and optimisation	^90^

## Conclusion

In conclusion, the trajectory of nanozyme research has fundamentally shifted from the serendipitous discovery of nanomaterials with intrinsic enzyme-like properties to the rational design of highly precise catalytic nanomachines. As elucidated in this review, their catalytic proficiency is not merely a mimicry of natural enzymes, but is intrinsically dictated by their unique nanoscale characteristics, surface defect engineering, and tuneable geometric configurations. By critically re-evaluating their mechanisms into distinct ROS-generating and electron-transfer pathways, and highlighting the structural evolution towards the atomic frontier—specifically single-atom and dual-atom nanozymes (SANs/DANs)—we provide a clearer, atomistic roadmap for their functional optimisation.

The integration of these fundamental theories has catalysed unprecedented advancements across the biomedical spectrum. From empowering ultra-sensitive, point-of-care platforms for in vitro diagnostics to executing precise in vivo interventions in synergistic tumour therapy and antibacterial strategies, nanozymes have proven their unparalleled versatility. Notably, their emerging ability to modulate cellular microenvironments and direct cell fate has opened highly promising avenues in regenerative medicine, including wound healing and vascular repair.

Nevertheless, realising their full clinical potential requires crossing a formidable translational chasm. Future endeavours must permanently move beyond empirical “trial-and-error” formulations. By embracing computational nanozymology (via AI and machine learning) to overcome the activity-specificity trade-off, strictly profiling their long-term in vivo biological identities (e.g., navigating the protein corona effect), and establishing globally recognised standardised assays, the field can effectively mitigate current regulatory and biosafety hurdles. Ultimately, through rigorous interdisciplinary collaboration spanning materials science, enzymology, and pathology, nanozymes are poised to become indispensable, programmable theranostic tools, driving the next generation of precision nanomedicine and personalised healthcare

## Data Availability

Data sharing not applicable – no new data generated
